# Mutation Severity of *FBXO43* Determines Spermatogenic Outcome and Informs Precision ART Strategies

**DOI:** 10.1155/humu/7905936

**Published:** 2026-08-12

**Authors:** Yanqin Xiao, Yutong He, Lanlan Meng, Huan Zhang, Chaofeng Tu, Yueqiu Tan, Liang Hu

**Affiliations:** ^1^ Institute of Reproductive and Stem Cell Engineering, NHC Key Laboratory of Human Stem Cell and Reproductive Engineering, Xiangya School of Basic Medical Science, Central South University, Changsha, China, csu.edu.cn; ^2^ Clinical Research Center for Reproduction and Genetics in Hunan Province, Reproductive and Genetic Hospital of CITIC-XIANGYA, Changsha, China, zxxyyy.cn

**Keywords:** aneuploidy, APC/C, *FBXO43*, macrozoospermia, male infertility

## Abstract

**Objective:**

FBXO43 is a known inhibitor of the anaphase‐promoting complex/cyclosome (APC/C), a key E3 ubiquitin ligase that controls meiotic cell cycle progression. However, how different *FBXO43* mutations affect APC/C inhibition and lead to divergent clinical phenotypes remains unclear. This study is aimed at clarifying how different *FBXO43* mutations produce divergent clinical phenotypes in male infertility and to investigate their preliminary molecular mechanisms, thereby providing evidence‐based guidance for precision‐assisted reproduction in affected individuals.

**Methods:**

Two infertile patients carrying distinct compound heterozygous *FBXO43* variants—missense mutations in a macrozoospermia case and truncating mutations in a nonobstructive azoospermia (NOA) case—were identified through whole‐exome sequencing. Sperm morphology, chromatin status, and aneuploidy were assessed, and a testicular biopsy was performed for the NOA case. Functional consequences of each mutation were evaluated using in vitro HEK 293T cell models, including protein stability, ubiquitination, and APC/C subunit interactions. Clinical outcomes of assisted reproductive technology (ART) were also reviewed.

**Results:**

Missense variants (NM_001029860, p.Pro641Leu/p.Arg660Gln) in the macrozoospermia patient allowed completion of meiosis but led to severe sperm head enlargement, chromatin condensation defects, and markedly elevated aneuploidy, resulting in repeated ICSI failure. In contrast, the truncating variants (p.Trp532 ^∗^/p.Ser577Leufs ^∗^11) in the NOA patient abolished the C‐terminal functional domain and caused meiotic arrest with complete absence of mature sperm. Mechanistically, all mutations reduced FBXO43 protein stability and disrupted its specific binding to the APC/C substrate recognition subunit APC3, whereas interactions with APC2/6/8 remained intact. Loss of APC3 binding likely impairs APC/C inhibition, disturbing meiotic chromosome segregation in a mutation‐severity–dependent manner.

**Conclusions:**

Different types of *FBXO43* mutations generate distinct infertility phenotypes through a dose‐dependent mechanism: Truncating mutations cause profound meiotic arrest and NOA, whereas hypomorphic missense mutations permit meiotic completion but result in severe teratozoospermia with high aneuploidy. These findings link FBXO43 to a continuous phenotypic spectrum and highlight its relevance for precision reproductive counseling. Based on the identified mutation‐specific risks, preimplantation genetic testing for aneuploidy (PGT‐A) is recommended for affected couples to improve embryo selection and optimize ART outcomes.

## 1. Introduction

Genetic etiologies account for approximately 15% of male infertility cases and represent a major cause of nonobstructive azoospermia (NOA) and severe spermatogenic failure [1] [2] [3]. According to the Human Phenotype Ontology (HPO) database, nearly 65 genes have been implicated in NOA, including meiotic regulators (e.g., *DMC1*), transcriptional regulators (e.g., *NR5A1*), and spermiogenesis‐related factors (e.g., *SPATA16*) [4]. Perturbations in any of these spermatogenic stages can arrest germ‐cell development and lead to a clinical presentation of NOA [5]. With advances in next‐generation sequencing, an increasing number of meiotic and spermatogenesis‐related genes have been implicated in human infertility, highlighting the importance of precise molecular diagnosis [6]. Whole‐exome sequencing (WES) and genome sequencing (GS), in particular, have been instrumental in uncovering the genetic etiology of idiopathic NOA [7], often by identifying disruptions in meiotic genes. Meiosis, a highly coordinated process essential for generating haploid gametes, requires accurate homologous recombination, chromosome segregation, and cell cycle control. Disruptions in these pathways often result in spermatogenic arrest, ultimately manifesting as clinical NOA [8]. Therefore, identifying genotype–phenotype correlations among meiosis‐related genes is critical for refining diagnostic workflows and informing assisted reproductive strategies.

Human spermatogenesis spans three major stages: mitotic proliferation, meiotic divisions, and spermiogenesis. Each stage is governed by complex transcriptional, epigenetic, and posttranslational regulatory networks [9] [10]. Among them, the ubiquitin (Ub)–proteasome system (UPS) plays a pivotal role in orchestrating protein turnover during meiotic progression and spermatid remodeling [11]. F‐box proteins, as substrate‐recognition components of the SCF (SKP1–CUL1–F‐box) Ub ligase complex, are key regulators of cell cycle transitions. Dysregulation of F‐box proteins such as FBXO5 (Emi1), FBXO47, and FBXO24 has been associated with mitotic defects, meiosis, and aberrant sperm morphology in human and animal models [12–14]. These observations underscore the indispensable role of protein degradation pathways in maintaining meiotic fidelity and genomic stability.

*FBXO43*, also known as early mitotic inhibitor 2 (Emi2) in murine models, is a crucial regulator of meiosis II exit through its inhibition of the anaphase‐promoting complex/cyclosome (APC/C) [15–17]. Emi2‐mediated APC/C suppression preserves cyclin B1 stability during oocyte maturation, and homologous pathways are thought to contribute to male germ‐cell meiotic control [18]. According to the TEDD (Temporal Expression during Development) database and the Human Testis Atlas, *FBXO43* transcripts are enriched in human spermatocytes and round spermatids [19, 20], aligning with its role as a meiotic regulator. However, only two independent studies have reported biallelic *FBXO43* variants causing male infertility yet with markedly distinct clinical presentations: the homozygous missense variant (p.Gly664Asp) leading to oligoasthenoteratozoospermia [21] and the homozygous truncating variant (p.Gln583 ^∗^) resulting in NOA with meiotic arrest [22]. These findings suggest that FBXO43 plays a critical role in human male meiosis; however, the extent to which different classes of mutations generate divergent phenotypes, and the underlying molecular mechanisms, remain largely unclear. This gap limits the interpretation of *FBXO43* variants in clinical diagnostics and hampers the development of mutation‐informed reproductive counseling.

This study is aimed at elucidating the genetic basis linking *FBXO43* mutations to divergent male infertility phenotypes by analyzing two unrelated patients harboring distinct compound heterozygous variants. Through WES, detailed morphological and cytological sperm assessment, and functional assays evaluating protein stability, ubiquitination, and APC/C interactions. By establishing mechanistic connections between *FBXO43* dysfunction and varying degrees of meiotic impairment, our findings provide novel insights into the genotype–phenotype continuum of *FBXO43*‐related male infertility and offer mutation‐specific recommendations to guide precision‐assisted reproduction strategies.

## 2. Materials and Methods

### 2.1. Human Subjects

A total of 118 patients diagnosed with asthenoteratospermia and 626 patients with NOA were recruited at the CITIC‐Xiangya Reproductive and Genetics Hospital from 2017 to 2023. All participants exhibited normal karyotypes. Semen analyses were performed following the World Health Organization Laboratory Manual for the Examination and Processing of Human Semen, fifth edition [23].

Azoospermia was diagnosed based on at least three independent semen analyses, with confirmation by the absence of spermatozoa after centrifugation and microscopic evaluation of semen pellets. Teratozoospermia was defined as < 4% morphologically normal spermatozoa. Written informed consent was obtained from all individuals. The study was conducted in accordance with protocols approved by the institutional ethics committees of CITIC‐Xiangya Reproductive and Genetics Hospital (LL‐SC‐2019‐034).

### 2.2. WES and Variant Filtering

Genomic DNA (gDNA) was extracted from peripheral blood using the QIAamp DNA Midi Kit (Qiagen, Hilden, Germany). WES libraries were constructed using the SureSelect Human All Exon V6 Kit (Agilent, Santa Clara, CA, USA) and sequenced on the Illumina HiSeq 2000 platform (Illumina, San Diego, CA, USA). Sequencing reads were subjected to standard quality‐control procedures and subsequently aligned to the human reference genome GRCh37/hg19 using the Burrows–Wheeler Aligner. Variant annotation was performed using ANNOVAR.

Candidate variants were prioritized based on the following criteria: (i) frequency filtering: retained heterozygous or homozygous variants with minor allele frequency < 0.05 in 1000 Genomes, gnomAD, and gnomAD‐EAS; (ii) functional impact prediction: retained variants predicted as deleterious by SIFT, MutationTaster, PolyPhen‐2, and CADD (CADD phred score ≥ 20); (iii) tissue‐specific expression: prioritized variants in genes with testis‐enriched or testis‐specific profiles based on the Human Protein Atlas, the Human Testis Atlas, and the NCBI Gene database; (iv) model organism evidence: further prioritized genes implicated in spermatogenesis or male infertility in animal knockout models; and (v) phenotype consistency: final variant selection required consistency between gene function and the patient′s clinical phenotypes [24].

In addition, we specifically examined a list of known pathogenic genes associated with NOA and asthenoteratospermia (e.g., *AURKC*, *SYCP3*, and *TEX11*) from the literature and ClinVar. Apart from the *FBXO43* variants identified in this study, no pathogenic or likely pathogenic variants were found in these known genes.

### 2.3. Preparation of Sperm Smears and Hematoxylin–Eosin (HE) Staining

Thawed semen samples were mixed with PBS at a 1:5 ratio and centrifuged at 3000 rpm for 5 min. Ten microliters of the sperm pellet suspension was smeared onto glass slides and air‐dried. Smears were fixed in 4% paraformaldehyde for 20 min and stored at −20°C or used immediately.

For HE staining, slides were rehydrated through graded ethanol (100%–50%), stained with hematoxylin for 10 min, differentiated in 10% acid ethanol, blued under running water, counterstained with eosin, dehydrated, cleared in ethanol–xylene, and mounted with neutral resin.

### 2.4. Aniline Blue Staining

Fixed sperm smears were incubated in aniline blue solution for 5 min at room temperature, rinsed thoroughly, dehydrated through 70%–100% graded ethanol, cleared in 1:1 ethanol–xylene solution, and mounted with neutral resin.

### 2.5. Transmission Electron Microscopy (TEM)

Semen samples were washed with 0.9% saline and fixed overnight at 4°C in 2.5% glutaraldehyde. Postfixation processing, ultrathin sectioning, and imaging were performed at the Cryo‐Electron Microscopy Center of Zhejiang University.

### 2.6. Fluorescence In Situ Hybridization (FISH)

Probes for chromosomes 18 (white), X (green), and Y (red) and for chromosomes 3 (red), 7 (green), and 15 (white) were purchased from Abbott‐Vysis, Inc. (Downers Grove, IL, USA). Fixed sperm smears were permeabilized with 0.05 M DTT, dehydrated through 70%–100% graded ethanol, and baked at 60°C. RNase A treatment was carried out at 37°C for 40 min. Probe and sample DNA were co‐denatured at 75°C for 5 min, followed by hybridization at 37°C overnight. Slides underwent stringent washing, counterstained with DAPI, and imaged under a fluorescence microscope as previously described [25]. For each FISH probe set, 200 spermatozoa were counted per sample.

### 2.7. Construction of Mutant Plasmids

Mutant *FBXO43* plasmids corresponding to the patient‐identified variants were generated using the Mut Express II Fast Mutagenesis Kit V2 (Vazyme, Jiangsu, China). Mutation‐specific primers were designed using the CE Design online tool (https://crm.vazyme.com/cetool/singlefragment.html). The primer sequences are listed in Table S1, and site‐directed mutagenesis was performed using Phanta Max high‐fidelity DNA polymerase. After PCR amplification, the PCR products were digested with DpnI, gel‐purified, and ligated via Exnase II–mediated homologous recombination. Recombinant plasmids were validated by Sanger sequencing.

### 2.8. Cell Culture and Transfection

HEK293T cells were cultured in DMEM (Gibco, Thermo Fisher Scientific, Waltham, MA, USA) supplemented with 10% fetal bovine serum (CellMax, Beijing, China) and 1% penicillin–streptomycin at 37°C in a humidified 5% CO₂ incubator. Cells were seeded at a density of 5 × 10^5^ cells per well into six‐well plates and transfected using the SuperKine Lipo3.0 reagent (Abbkine, Hubei, China) with 3 *μ*g plasmid DNA per well. The medium was refreshed after 8 h, and cells were harvested 48 h posttransfection.

### 2.9. Immunofluorescence

Cells were seeded onto coverslips, fixed with 4% paraformaldehyde, permeabilized with 0.5% Triton X‐100, blocked, and incubated overnight at 4°C with an anti‐FLAG antibody (1 *μ*g/mL, GenScript, Jiangsu, China). After washing, fluorescent secondary antibodies (1:100) were applied for 1 h at room temperature in the dark, followed by DAPI counterstaining. Coverslips were mounted with an anti‐fade reagent and imaged under a fluorescence microscope.

### 2.10. Western Blotting

Cells were lysed in RIPA buffer supplemented with protease inhibitors. Lysates were denatured, separated on SDS–PAGE gels, transferred onto PVDF membranes, blocked in 5% skim milk, and incubated overnight at 4°C with anti‐FLAG or anti‐HA primary antibodies (0.1 *μ*g/mL, GenScript, Jiangsu, China). After washing with TBST, membranes were incubated with HRP‐conjugated secondary antibodies (1:50,000) at room temperature for 1 h. Protein bands were visualized using enhanced chemiluminescence (ECL) reagents and quantified using ImageJ.

### 2.11. Immunoprecipitation–Mass Spectrometry

HEK293T cells transfected with the indicated plasmids were lysed, and immune complexes were captured with magnetic beads, washed with TEAB buffer, and digested with 500‐ng trypsin at 37°C for 4 h, followed by an additional 500‐ng trypsin for overnight digestion. Peptides were desalted online using a PEPMAP trap column (Thermo Fisher Scientific, Waltham, MA, USA) and separated on a PepMap analytical column (Thermo Fisher Scientific, Waltham, MA, USA) with a gradient of 3%–38% solvent B (80% acetonitrile, 0.1% formic acid) over 102 min. Eluted peptides were analyzed using an Orbitrap mass spectrometer coupled with a FAIMS interface (compensation voltages: −35, −45, and−65 V), operated at a resolution of 120,000 for MS1 with a 40‐s dynamic exclusion; MS2 spectra were acquired in the linear ion trap. Raw data were processed using Proteome Discoverer 2.5 (San Jose, CA) and searched against the 2022 UniProt human database with precursor and fragment tolerances of 10 ppm and 0.6 Da, respectively. Search parameters included a peptide length of 6–144 amino acids and up to two missed cleavages, and proteins were filtered at a false discovery rate (FDR) ≤ 0.01.

### 2.12. Statistical Analysis

All experiments were performed in at least three independent biological replicates unless otherwise specified. Quantitative data are presented as mean ± standard deviation (SD). Statistical analyses were conducted using GraphPad Prism v8.0 (GraphPad Software, San Diego, CA, USA). Comparisons between two groups were performed using two‐tailed unpaired Student′s *t* tests. A *p* value of < 0.05 was considered statistically significant.

### 2.13. Addressing Potential Bias

Several steps were taken to minimize potential biases in this study. Selection bias was acknowledged as inherent in the study of rare genetic variants; our cases were systematically identified from a larger cohort of idiopathic infertile men undergoing genetic screening. Measurement and observer biases were mitigated by using standardized, protocol‐driven laboratory techniques for all assays. Confounding by other genetic factors was addressed by using WES to exclude pathogenic variants in other known genes associated with spermatogenic failure.

## 3. Result

### 3.1. Identification of Pathogenic *FBXO43* Mutations in Two Affected Patients

We performed WES on 118 patients with asthenoteratospermia and 626 patients with NOA, within which the two probands analyzed in this study (P1‐II‐1 and P2‐II‐1) were included. According to our previously described stringent variant‐filtering strategy , we identified four rare variants in *FBXO43* across these two unrelated families.

**Figure 1:**
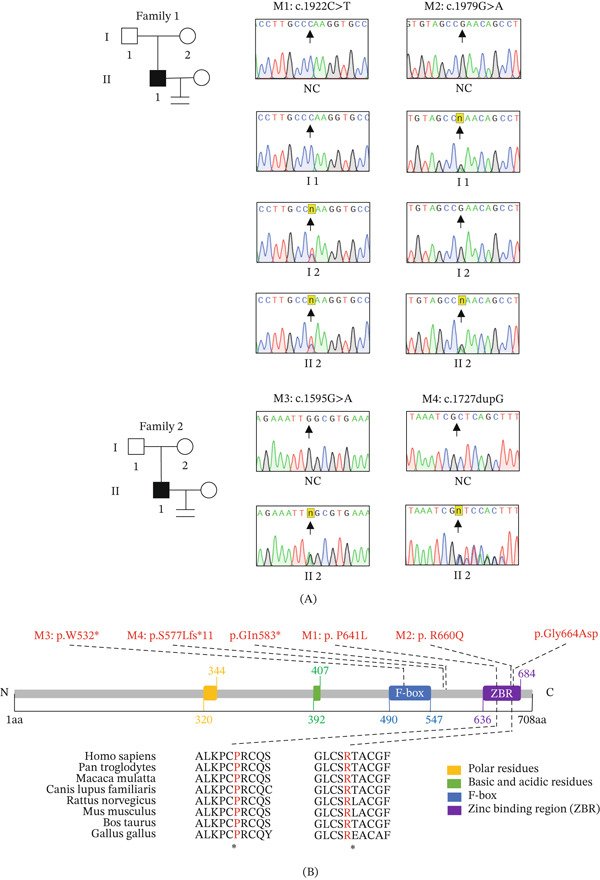
Identification of *FBXO43* variants in two unrelated patients. (A) Sanger sequencing validation of *FBXO43* variants in P1 and P2. In P1, each missense variant was inherited from one parent (arrows), consistent with an autosomal recessive pattern. Parental samples from P2 were unavailable, precluding inheritance determination. Black arrows indicate the variant positions. (B) Schematic representation of FBXO43 protein secondary structure. Amino acid sequences were retrieved from UniProt. The two missense variants in P1 occur at highly conserved residues across multiple species. Conservation analysis was not performed for the nonsense and frameshift variants identified in P2.

In Family 1 (P1), WES revealed two compound heterozygous missense variants in *FBXO43* (M1: p.Pro641Leu; M2: p.Arg660Gln). Sanger sequencing confirmed biparental inheritance of the two alleles, supporting an autosomal recessive mode. In Family 2 (P2), the affected individual carried one nonsense variant (M3: p.Trp532*) and one frameshift variant (M4: p.Ser577Leufs ^∗^11). Because parental samples were unavailable, the mode of inheritance could not be determined (Figure 1A).

All four variants are exceedingly rare in population databases (allele frequency < 1%) and are consistently predicted to be deleterious by multiple in silico algorithms, supporting their pathogenic potential (Table 1). The two missense variants in P1 localize in the evolutionarily conserved ZBR domain, whereas the truncating mutations in P2 are predicted to abolish the ZBR domain entirely, thereby likely resulting in substantial loss of FBXO43 function (Figure 1B). For comparison, two previously reported *FBXO43* variants—the missense variant p.Gly664Asp (associated with oligoasthenoteratozoospermia) and the truncating variant p.Gln583* (associated with NOA)—are also mapped onto the domain schematic in Figure 1B. *FBXO43* has been identified as a meiosis‐associated male infertility gene based on evidence from mouse knockout models and human case reports [22, 26]. Taken together, these data support that pathogenic variants in *FBXO43* represent the shared underlying genetic etiology of male infertility in both probands.

**Table 1:** *FBXO43* gene variant information.

Patient	CDNA variant	Amino acid change	Genotype	Variant type	1000 G^a^	ExAC_ALL^b^	ExAC_EAS^c^	SIFT	Mutation taster	PolyPhen‐2	CADD^d^
P1	M1: c.1922C > T	p.P641L	Hetero	Missense	0	0.00007	0.0003	D	D	D	33
	M2: c.1979G > A	p.R660Q	Hetero	Missense	0	0	0	D	D	D	32
P2	M3: c.1595G > A	p.W532 ^∗^	Hetero	Nonsense	0	0	0	D	D	NA	NA
	M4: c.1727dupG	p.S577Lfs ^∗^11	Hetero	Frameshift	0	0	0	D	D	NA	45

*Note:* D indicates damaging, referring to variants predicted to be deleterious; NA indicates that the software is unable to generate predictions for frameshift or nonsense variants.

^a^Frequency in the 1000 Genomes Project.

^b^Frequency in the Exome Aggregation Consortium dataset.

^c^Frequency in the East Asian population subgroup of the Exome Aggregation Consortium dataset.

^d^CADD score; substitutions with a score > 4 are predicted to be deleterious.

### 3.2. Clinical Characteristics of the Patients

Two affected probands from unrelated Han Chinese families were diagnosed with severe primary male infertility. Both individuals exhibited normal somatic karyotypes and no detectable Y‐chromosome microdeletions, suggesting that their conditions were unlikely to result from known chromosomal abnormalities or AZF microdeletion.

Proband P1 presented with macrozoospermia. Semen analysis indicated largely preserved spermatogenic output, with ejaculate volume and sperm concentration within the WHO reference range. However, sperm functional parameters were profoundly impaired. The proportion of progressively motile sperm was only 13.56%, and the percentage of morphologically normal spermatozoa was 3.3% (Table 2). The patient had a 10‐year history of infertility and had undergone seven ICSI cycles at five reproductive centers. In total, 19 embryos were transferred. Only one transfer resulted in a clinical pregnancy, which subsequently ended in early miscarriage, whereas all remaining embryos failed to implant (Table 3). This consistent pattern across repeated cycles and hospitals suggests that the sperm defects likely extended beyond abnormalities by routine semen analysis and interfered with crucial processes during early embryonic development, ultimately impairing embryonic developmental potential.

**Table 2:** Semen parameters of the macrozoospermia patient (P1).

Clinical characteristics		P1	Reference value
Testicular volume	Left (mL)	13.3	10–15
	Right (mL)	13.6	
Semen analysis	Sperm concentration (10^6^/mL)	27.88	> 15
	Semen volume(mL)	5.1	> 1.5
	Progressive motility (%)	13.56	> 32%
	Normal sperm morphology(%)	3.3	> 4%
Karyotype		46, XY	46, XY
Y chromosome microdeletion		No	No

**Table 3:** Assisted reproductive technology (ART) information for the macrozoospermia patient (P1).

Clinical indicators	Hospital 1			Hospital 2		Hospital 3		Hospital 4	
ART cycle no.	1		2	3	4	5		6	7
Male age	24		24	26	27	29		33	35
Female age	25		25	27	28	30		34	36
Number of oocytes retrieved	24		9	12	12	24		8	1
Fertilized eggs	NA		NA	NA	NA	NA		6	1
Total embryos	6		3	3	3	4		6	1
Eight‐cell embryos	NA		NA	NA	NA	NA		2	0
Blastocysts	NA		NA	NA	NA	NA		1	0
Transfer cycle	1	2	1	1	1	1	2	1	—
Number of embryos transferred	2	3	3	3	3	2	2	1	—
Successful implantation	No	No	No	No	No	No	No	Yes	—
Clinical outcome	NP	NP	NP	NP	NP	NP	NP	Miscarriage	TC

*Note:* Fertilization method: All cycles were performed using intracytoplasmic sperm injection (ICSI). Ovarian reserve indicators (AMH and AFC) and detailed embryo quality grades were not uniformly documented across the five reproductive centers; therefore, only the numbers of oocytes retrieved, fertilized eggs, total embryos, eight‐cell embryos, and blastocysts are presented where available.

Abbreviations: NA, not available (data not recorded by the treating center); NP, not pregnant; TC, transfer cancelled.

**Figure 2:**
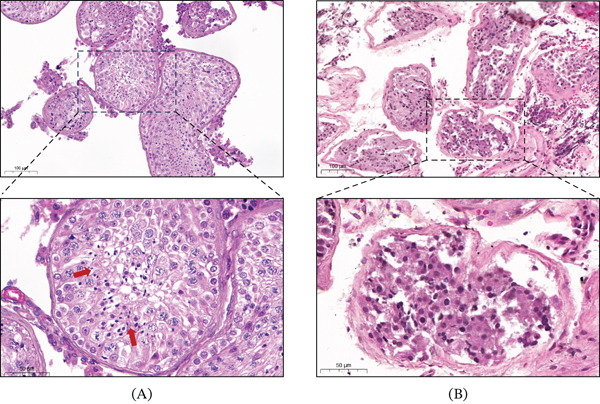
Histological analysis of testicular tissue from the NOA patient (P2). (A) HE‐stained testicular section from a control patient with obstructive azoospermia (OA). Spermatogenic cells are arranged along the basal compartment, and elongated spermatids are present within the seminiferous tubular lumen (red arrows). (B) HE‐stained testicular section from patient P2. Seminiferous tubules show marked degeneration and architectural distortion. Germ cells are present, but no postmeiotic spermatids or spermatozoa are observed.

Proband P2, who originated from a consanguineous family, exhibited azoospermia. Physical examination revealed bilateral testicular volumes of approximately 8–10 mL, which is below the adult reference range (10–15 mL). Three consecutive semen analyses performed at our hospital showed azoospermia upon centrifuged pellet examination. To further assess spermatogenic function, a diagnostic testicular puncture biopsy was performed. Histopathological analysis revealed narrowed seminiferous tubule diameter, disorganized tubular wall architecture, and degenerative spermatogenic function. Although primary spermatocytes were present, no spermatids were detected, consistent with a histological picture suggestive of meiotic arrest. (Figure 2). These findings collectively indicated an extremely low likelihood of endogenous sperm production. The patient ultimately proceeded with donor‐sperm insemination.

### 3.3. Aneuploidy and Impaired Chromatin Condensation in Proband P1 Sperm

**Figure 3:**
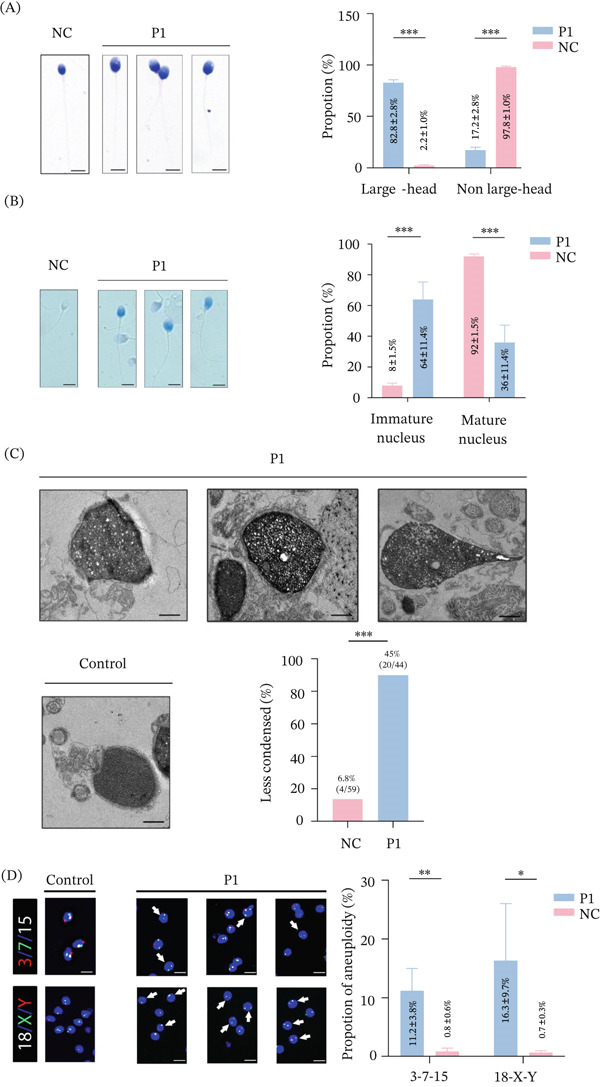
Sperm morphology and chromatin defects in patient P1. (A) HE staining and quantification of macrocephalic sperm. (B) Aniline blue staining and quantification of immature sperm nuclei. Percentages represent the proportions of mature and immature spermatozoa in P1 and control samples. (C) Transmission electron microscopy of P1 sperm nuclei, showing nuclear vacuolization and reduced condensation. Due to limited sample availability, statistical comparison was not performed. (D) Fluorescence in situ hybridization (FISH) analysis of sperm aneuploidy using two sets of chromosome‐specific probes to assess the proportion of aneuploid sperm in P1 and controls. For each FISH probe set, 200 spermatozoa were counted per sample. Scale bars: A, B, D = 10 *μ*m; C = 2 *μ*m.  ^∗^ *p* < 0.05,  ^∗∗^ *p* < 0.01,  ^∗∗∗^ *p* < 0.001.

To characterize the morphological abnormalities in P1 sperm, HE staining was performed. Proband P1 exhibited an extremely high proportion of abnormal sperm heads (98.7%), of which 82.8 ± 2.8% were macrocephalic, a rate markedly elevated compared with controls (2.2 ± 1.0%, *p* < 0.0001) (Figure 3A). Tail morphology appeared normal.

Given that macrocephalic sperm are frequently associated with chromatin‐remodeling defects, we next evaluate nuclear maturation using aniline blue staining. A substantial proportion of P1 spermatozoa retained histones, with 64 ± 11.4% staining positive, compared with 8 ± 1.5% in controls (*p* < 0.0001) (Figure 3B), indicating impaired histone‐to‐protamine replacement. Ultrastructural analysis by TEM further revealed disorganized chromatin architecture, reduced nuclear condensation, and prominent vacuolization (Figure 3C).

To assess chromosomal integrity, FISH analysis using probes for chromosomes 3, 7, 15, 18, X, and Y revealed a significantly elevated aneuploidy rate in P1 sperm (chromosomes 3/7/15: 11.2 ± 3.8%; chromosomes 18/X/Y: 16.3 ± 9.7%) (Figure 3D), indicating aberrant meiotic chromosome segregation.

Together, these findings demonstrate that sperm from P1 exhibit a typical macrozoospermia phenotype accompanied by impaired nuclear maturation, defective chromatin condensation, and chromosome segregation errors, collectively indicating severe defects in meiotic progression and chromatin remodeling.

### 3.4. FBXO43 Variants Reduce Protein Stability Via Enhanced Ubiquitination

To investigate the structural impact of patient‐derived FBXO43 variants, we retrieved the predicted two‐dimensional protein structure from AlphaFold (https://alphafold.com/)and modeled amino acid substitutions using the Mutagenesis Wizard (V.1.5) in PyMOL. Structure simulation of the P1 missense variant p.Pro641Leu (M1) revealed that the Pro‐to‐Leu substitution does not overtly disrupt protein folding (Figure 4A). Notably, Pro641 resides in the *α*‐helical region of the ZBR (Zinc Binding Region) domain, positioned at the junction of two helices. Thus, this residue may contribute to structural rigidity and domain stability through its conformational role. Modeling the second P1 variant, p.Arg660Gln (M2), showed loss of a hydrogen bond between Arg660 and Ser645 (Figure 4B). Because hydrogen bonding is important to stabilize local protein structure, this disruption may compromise protein stability.

Figure 4Structural prediction of FBXO43 variants and functional validation through ubiquitin‐mediated degradation. (A) Predicted hydrogen‐bonding interactions surrounding residue Pro641 in wild‐type FBXO43 and after mutation. (B) Predicted hydrogen‐bonding interactions surrounding residue Arg660 in wild‐type FBXO43 and after mutation. In each panel, the upper row shows the wild‐type residue and the lower row shows the mutant residue. The ZBR domain is shown in blue, the F‐box domain in brown, mutated residues in red, residues involved in hydrogen bonding in green, and hydrogen bonds are indicated by yellow dashed lines. (C) Protein expression analysis 48 h after transfection into HEK293T cells. Three independent transfection replicates were performed, and protein levels were quantified using ImageJ (two‐way ANOVA).  ^∗^ *p* < 0.05,  ^∗∗^ *p* < 0.01,  ^∗∗^ *p* < 0.001. The p.Gly664Asp variant, previously reported in a patient with oligoasthenoteratozoospermia, is included for comparison. All FBXO43 mutations result in reduced protein abundance. (D) Assessment of FBXO43 ubiquitination following cotransfection of FBXO43 and Ub plasmids into HEK293T cells. Immunoprecipitation was performed 48 h posttransfection. Mutant proteins show mildly increased ubiquitination compared with wild‐type FBXO43. High ubiquitination signals in the control group likely reflect elevated Ub expression and nonspecific binding to the FLAG antibody.

**Figure:**
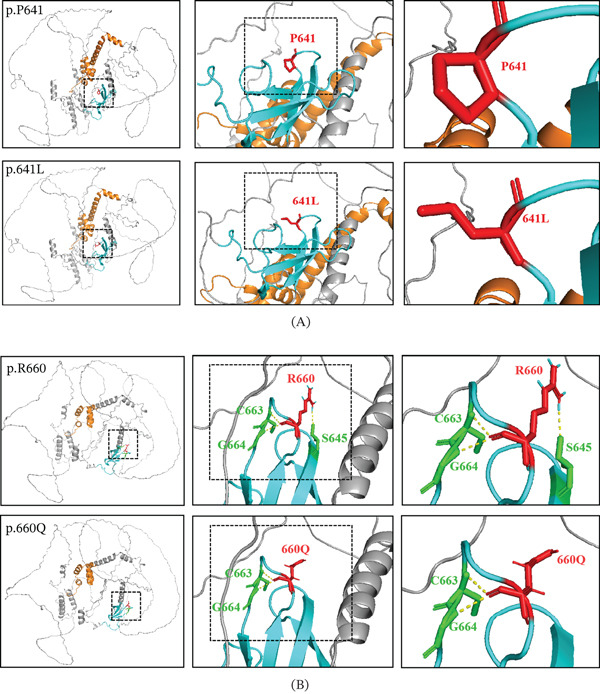

**Figure:**
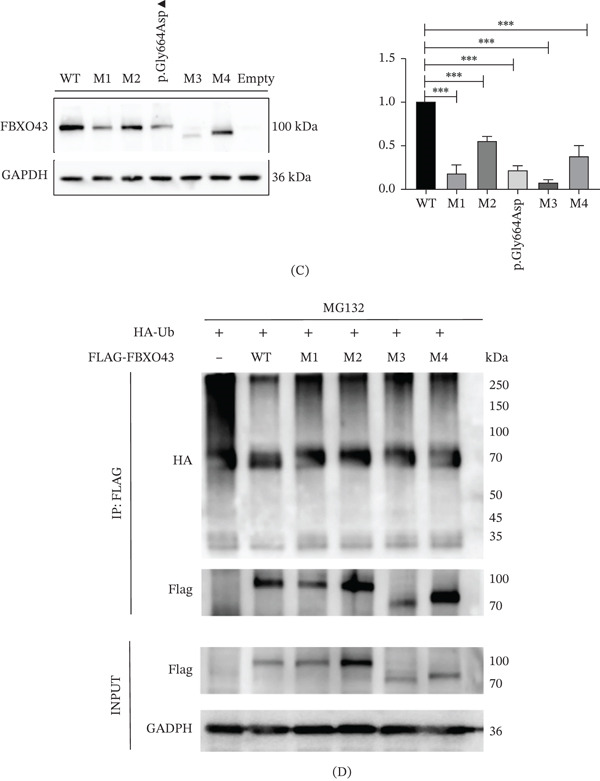

To experimentally evaluate the functional impact of FBXO43 mutations, N‐terminal 3 × FLAG‐tagged wild‐type and mutant FBXO43 constructs (M1: p.Pro641Leu; M2: p.Arg660Gln; M3: p.Trp532*; M4: p.Ser577Leufs ^∗^11) were expressed in HEK293T cells. Both missense variants (M1 and M2) exhibited significantly reduced protein abundance (approximately 80% and 44% of wild‐type, respectively; *p* < 0.001), consistent with our hypothesis that mutations within key residues of the ZBR domain destabilize the protein and/or promote degradation (Figure 4A). The truncating variants M3 and M4 displayed electrophoretic mobility and markedly reduced expression (~90% and 60% loss, respectively; *p* < 0.001), consistent with predicted C‐terminal truncation. For comparison, we included the previously reported homozygous missense variant p.Gly664Asp, which is associated with oligoasthenoteratozoospermia [21]. No functional studies for this variant were included in the original report. It represents a hypomorphic allele that permits meiotic completion, with functional severity intermediate between our P1 missense variants and P2 truncating mutations. In our assay, this variant yielded an intermediate reduction in protein abundance (77%) (Figure 4C). These findings suggest that the FBXO43 protein dosage may negatively correlate with phenotypic severity.

Given prior evidence that fertilization‐triggered Ca^2+^ influx activates FBXO43 phosphorylation and subsequent SCF*β*–TrCP–mediated ubiquitination and proteasomal degradation [27], we next assess ubiquitination levels by co‐transfecting FBXO43 constructs with Ub plasmids. All mutants exhibited enhanced ubiquitination relative to wild‐type (Figure 4D), indicating that reduced protein abundance results from accelerated proteasomal degradation. Immunofluorescence analysis showed that both wild‐type and mutant FBXO43 predominantly localized to the cytoplasm with a weak nuclear signal and displayed a diffuse perinuclear distribution pattern (Figure S1), suggesting that subcellular localization was not affected by the variants. Collectively, these results indicate that FBXO43 variants impaired protein stability via increased Ub‐mediated degradation rather than mislocalization, providing a mechanistic explanation for the reduced protein levels observed across patient‐derived mutations.

### 3.5. *FBXO43* Mutations Disrupting its Specific Interaction With APC/C Subunits Impair Meiosis and Cause Spermatogenic Failure

**Figure 5:**
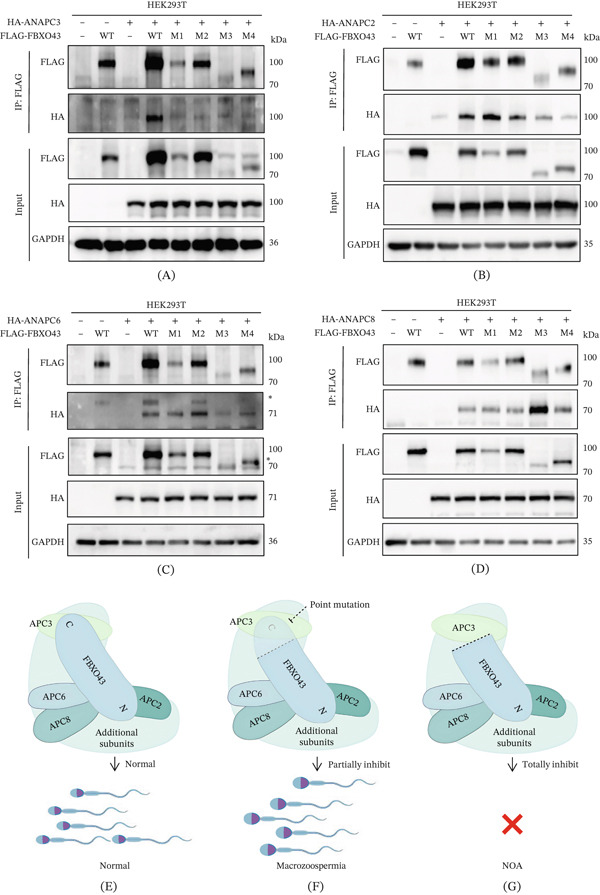
Interaction of FBXO43 with APC/C subunits. (A) Co‐immunoprecipitation (Co‐IP) analysis following cotransfection of FBXO43 with ANAPC3. Wild‐type FBXO43 strongly interacts with APC3, whereas patient‐derived mutations attenuate this interaction. (B) Co‐IP analysis following cotransfection of FBXO43 with ANAPC2. *FBXO43* mutations do not affect binding to APC2. (C) Co‐IP analysis following cotransfection of *FBXO43* with *ANAPC6*. FBXO43–APC6 interactions are preserved across all variants. ( ^∗^ indicates nonspecific bands.) (D) Co‐IP analysis following cotransfection of *FBXO43* with *ANAPC8*. *FBXO43* mutations do not impair binding to APC8. (E–G) Schematic model illustrating the functional consequences of *FBXO43* mutations. (E) Full‐length FBXO43 interacts with APC2, APC6, and APC8, whereas C‐terminal binding to APC3 inhibits APC/C activity, supporting normal spermatogenesis. (F) C‐terminal missense mutations weaken APC3 binding, resulting in macrozoospermia. (G) Loss‐of‐function mutations abolish APC3 binding and FBXO43‐mediated APC/C inhibition, leading to NOA. Other APC/C subunits are not shown.

To elucidate the molecular mechanisms by which *FBXO43* mutations affect chromosome segregation, we performed immunoprecipitation followed by mass spectrometry (IP‐MS) to identify the FBXO43 interactome. Due to the lack of suitable antibodies, FLAG‐tagged wild‐type FBXO43 was expressed in HEK293T cells for affinity enrichment. IP‐MS analysis revealed interactions between FBXO43 and seven core APC/C subunits (Table S2). Subsequent co‐immunoprecipitation validated that wild‐type FBXO43 specifically binded to APC3, a crucial APC/C substrate recognition subunit (Figure 5A), whereas all patient‐derived mutants (M1–M4) failed to maintain this interaction. According to the *Human Testis Atlas*, FBXO43 and APC3 are both enriched in human spermatocytes and round spermatids (Figure S3), supporting their functional interaction. Given that APC3 is directly involved in substrate recognition, the loss of FBXO43–APC3 binding suggests that FBXO43 may act as a “pseudo‐substrate” that competitively inhibits APC/C activity. Abrogation of this interaction is therefore likely to release APC/C inhibition, leading to APC/C dysregulation or premature activation. In contrast, both wild‐type and mutant FBXO43 retain interactions with other APC/C components, including APC2, APC6, and APC8 (Figure 5B–D). These subunits likely serve as structural anchors rather than direct functional regulators. No direct interactions were detected with other subunits, such as APC10, APC15, and APC16, as shown in Figure S2.

Collectively, these findings support a dual regulatory model in which FBXO43 modulates APC/C activity by functionally inhibiting APC3 and by maintaining structural anchoring via at least APC2, APC6, and APC8. Mutations that disrupt APC3 binding lead to a loss of inhibitory function, resulting in APC/C hyperactivation. This mechanistic framework provides insight into phenotype–genotype correlations of *FBXO43*: nonsense or frameshift mutations (e.g., M3/M4) that completely abolish APC3 interaction may cause severe meiotic arrest, manifesting as NOA, whereas missense mutations (e.g., M1/M2) that partially compromise protein stability but preserve some structural interactions may permit meiotic completion yet impair postmeiotic spermatogenesis, resulting in macrozoospermia (Figure 5E–G).

## 4. Discussion

Genetic defects affecting human meiosis often present as discrete clinical entities like NOA or severe oligoasthenoteratozoospermia, yet the biological processes underlying these diagnoses are inherently continuous. In this study, by integrating detailed clinical phenotyping with molecular analyses, we demonstrate that distinct classes of *FBXO43* mutations generate a spectrum of spermatogenic impairment, ranging from probable meiotic arrest based on histology to aberrant meiotic completion with profound chromosomal and chromatin defects. This continuum provides a unifying framework for understanding clinical observations and positions *FBXO43* as a dosage‐sensitive regulator of human male meiosis.

Previous animal and human studies have shown that severe loss‐of‐function variants in *FBXO43* are associated with meiotic arrest at metaphase I, resulting in NOA [22, 26]. In mice, homozygous *Emi2/FBXO43* knockout leads to meiotic arrest at the early diplotene stage and complete azoospermia, phenocopying human NOA patients with truncating *FBXO43* variants. The patient P2 carrying nonsense and frameshift variants (p.Trp532 ^∗^ and p.Ser577Leufs ^∗^11) exhibited NOA. Although pathological analysis was not feasible, the predicted loss of the F‐box and ZBR domains and markedly reduced protein abundance support a loss‐of‐function mechanism comparable with that reported for other truncating *FBXO43* variants. These observations reinforce the requirement of intact FBXO43 function for meiotic progression. In contrast, the macrozoospermia phenotype observed in human patients carrying hypomorphic missense variants (e.g., p.Pro641Leu/p.Arg660Gln in this study), as well as the oligoasthenoteratozoospermia phenotype associated with p.Gly664Asp [21], both of which permit meiotic completion, has not been reproduced in mouse models, likely because existing mouse models are complete knockouts rather than hypomorphic alleles that retain partial protein function. Future generation of knock‐in mice carrying patient‐equivalent missense mutations will be valuable to fully recapitulate the human phenotypic spectrum.

In the present study, compound heterozygous missense variants were associated with macrocephalic spermatozoa accompanied by defective chromatin condensation and markedly elevated aneuploidy, despite preserved sperm counts. Notably, a previously reported homozygous missense variant p.Gly664Asp [21] also permits meiotic completion but leads to severe sperm defects, indicating that partial FBXO43 dysfunction does not result in a single uniform phenotype. Rather, meiotic gene variants that allow completion of meiosis may manifest clinically as different forms of severe sperm dysfunction, including teratozoospermia as well as oligoasthenoteratozoospermia. Functional analyses in our study consistently show mutation‐dependent reductions in FBXO43 protein abundance, supporting the notion that residual protein dosage modulates both the severity and qualitative nature of postmeiotic defects. According to the ACMG/AMP 2015 guidelines, we classified the four *FBXO43* variants by integrating population frequency, inheritance, and experimental data. The truncating variants in P2 met PVS1 and PM2, supporting a pathogenic classification. For the missense variants in P1, the combination of PM2, PM3, and PP3 criteria, together with PS3_Supporting evidence from our functional assays, supported an upgrade to likely pathogenic. These classifications provide a genetic basis for the observed phenotypes and underscore the importance of functional validation in reproductive genes.

Mechanistically, FBXO43 functions as an inhibitor of anaphase‐promoting complex/cyclosome (APC/C), an E3 Ub ligase that regulates chromosome segregation during the metaphase‐to‐anaphase transition through timely substrate ubiquitination and degradation [28][29][30]. We demonstrated that wild‐type FBXO43 interacts with multiple APC/C subunits (APC3, APC2, APC6, and APC8), with a specific association with APC3, whereas patient‐derived variant proteins show markedly reduced or absent binding. FBXO43 likely acts as a “pseudo‐substrate” to inhibit APC/C, preventing premature degradation of key substrates such as Cyclin B1. Loss of FBXO43 function disrupts the temporal activation of APC/C, impairing the metaphase‐to‐anaphase transition, consistent with the observed sperm aneuploidy.

In addition to chromosome mis‐segregation, spermatozoa from the macrozoospermia patient exhibited pronounced defects in chromatin condensation and histone retention, suggesting impaired coordination between meiotic progression and nuclear remodeling. However, the molecular links between APC/C dysregulation and chromatin remodeling remain unclear. Subcellular localization studies in HEK293T cells showed predominantly cytoplasmic FBXO43, contrasting with nuclear localization reported in mouse spermatocytes and HCCLM3 cells [26][31], likely reflecting cell‐type‐specific regulatory contexts.

Clinically, meiotic disruptions resulting from *FBXO43* mutations adversely affect the success rates of assisted reproductive technology (ART). The macrozoospermia patient experienced repeated ICSI failure and early pregnancy loss, consistent with the high sperm aneuploidy detected in our study and prior reports [21] [32]. These findings underscore the limitations of conventional semen parameters in predicting reproductive competence and support the inclusion of meiosis‐related genes such as *FBXO43* in genetic evaluation of severe male infertility. In cases with elevated gamete aneuploidy, preimplantation genetic testing for aneuploidy (PGT‐A) may be considered when residual reproductive potential is present. For carriers of loss‐of‐function variants with poor reproductive outcomes, donor gametes may be preferable.

Several limitations define the scope of this study. All functional assays (protein stability, ubiquitination, and co‐immunoprecipitation) were performed in HEK293T cells, a heterologous system that does not fully recapitulate the native meiotic environment of human germ cells, and the small number of affected individuals limits quantitative genotype–phenotype modeling. Nevertheless, the clinical, cytological, and molecular evidence of our study may support that different *FBXO43* variants exert different effects on meiotic progression, giving rise to a continuous phenotypic spectrum of male infertility. This dosage‐dependent framework provides a rational basis for interpreting *FBXO43* variants in clinical diagnostics and for guiding individualized reproductive counseling.

## Author Contributions

Conceptualization: Yanqin Xiao, Liang Hu. Methodology: Yanqin Xiao, Yutong He, Liang Hu; Formal analysis and investigation: Yanqin Xiao, Yutong He, Lanlan Meng, Chaofeng Tu. Writing—original draft: Yanqin Xiao, Yutong He. Writing—review and editing: Liang Hu. Visualization: Yanqin Xiao. Funding acquisition: Liang Hu. Resources: Huan Zhang, Yueqiu Tan, Liang Hu. Supervision: Liang Hu. Yanqin Xiao and Yutong He contributed equally to this work.

## Funding

This study was supported by the Graduate Innovation Project of Central South University (2025ZZTS0943), Natural Science Foundation of Hunan Province (10.13039/501100004735, 2024JJ9087), and Hunan Provincial Grant for Innovative Province Construction (2019SK4012).

## Ethics Statement

This study was approved by the Ethics Committee of the Reproductive and Genetic Hospital of CITIC‐Xiangya of Central South University.

## Conflicts of Interest

The authors declare no conflicts of interest.

## Supporting Information

Additional supporting information can be found online in the Supporting Information section.

## Supporting information


**Supporting Information 1** Table S1. Primer sequences for *FBXO43* wild‐type and mutants. Table S2. APC/C subunits identified by IP–MS usingwild‐type FBXO43.

**Supporting Information 2** Figure S1. Immunofluorescence showing cytoplasmic localization of FBXO43 in HEK 293T cells, with no localization changes among wild‐type and mutants. Supplementary Figure S2. Co‐immunoprecipitation analysis showing no direct interaction between FBXO43 and ANAPC10, ANAPC15, or ANAPC16. Supplementary Figure S3. Cell‐type‐specific expression profiles of FBXO43 and APC3 in human testis, showing overlapping enrichment in spermatocytes and round spermatids.

## Data Availability

The data underlying this article will be shared on reasonable request to the corresponding author.
