# Supplementary material for: Mutation Severity of FBXO43 Determines Spermatogenic Outcome and Informs Precision ART Strategies
Source: Hum Mutat. 2026 Aug 12;2026:7905936. doi: 10.1155/humu/7905936 (PMC13469765; doi:10.1155/humu/7905936)
Supplement: Supplementary file 1 [file HUMU-2026-7905936-s002.zip › Supplementary Figures.pdf]

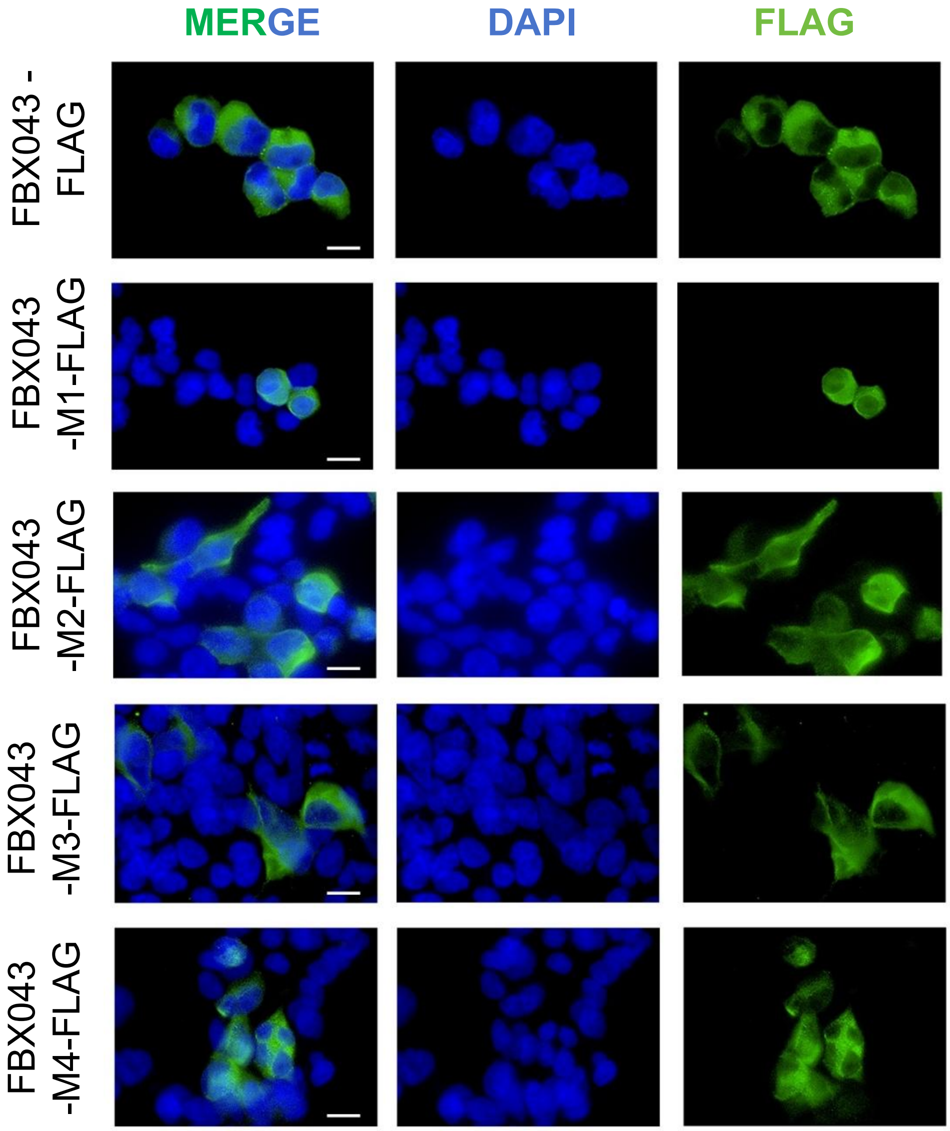

Supplementary Figure 1. Subcellular localization of FBXO43 in HEK 293T cells.

HEK 293T cells were transfected with FBXO43-expressing plasmids for 24 h and subsequently subjected to immunofluorescence staining to assess the subcellular localization of FBXO43. FBXO43 was predominantly localized in the cytoplasm. Representative images suggest no obvious change in localization pattern among wild-type and mutant FBXO43.

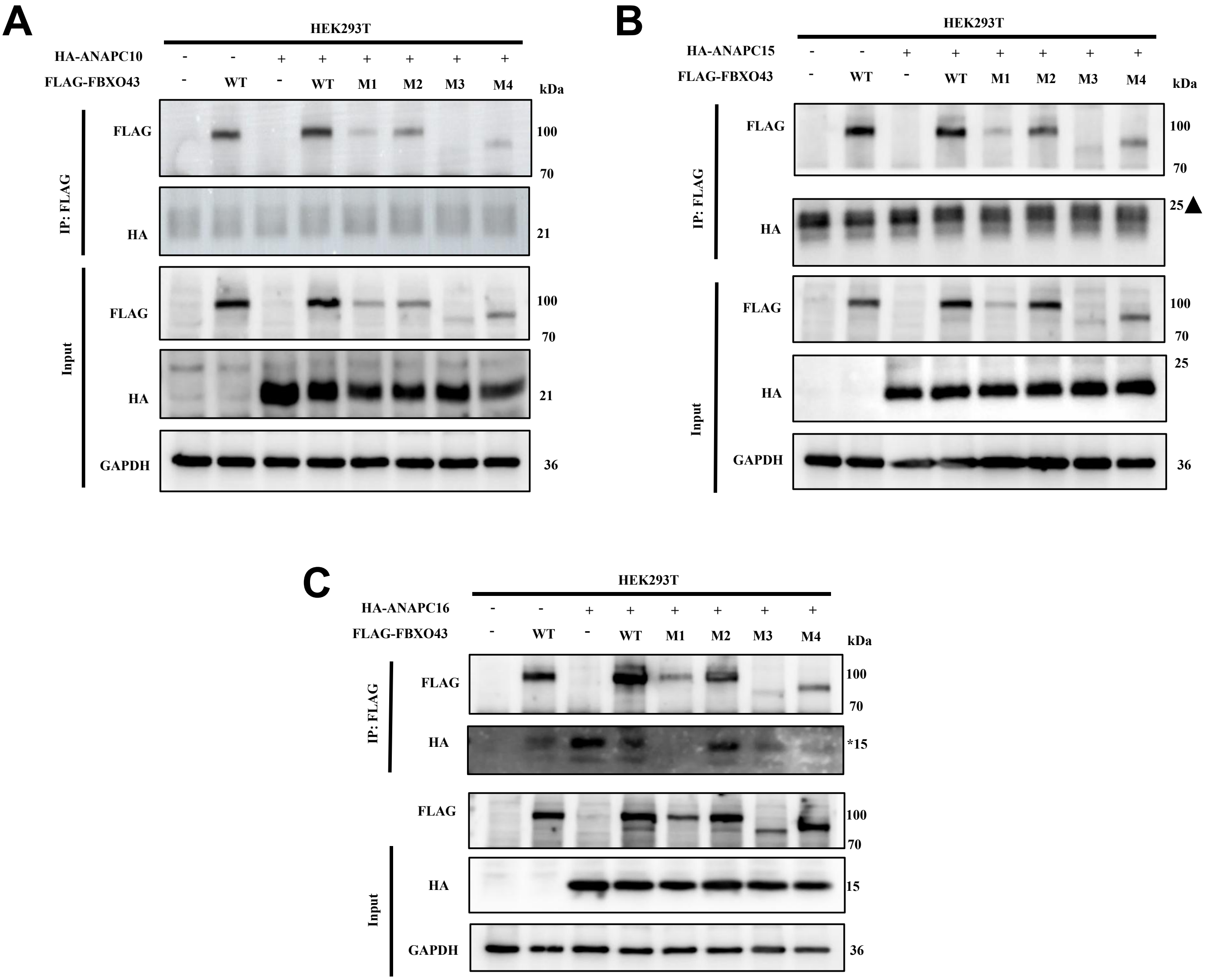

Supplementary Figure 2. Lack of direct interaction between human FBXO43 and some APC/C subunits.

(A) Co-immunoprecipitation (Co-IP) analysis following co-transfection of FBXO43 and ANAPC10. (B) Co-IP analysis following co-transfection of FBXO43 and ANAPC15. (C) Co-IP analysis following co-transfection of FBXO43 and ANAPC16. ▲ indicates the immunoglobulin light chain; * denotes nonspecifically bound bands.

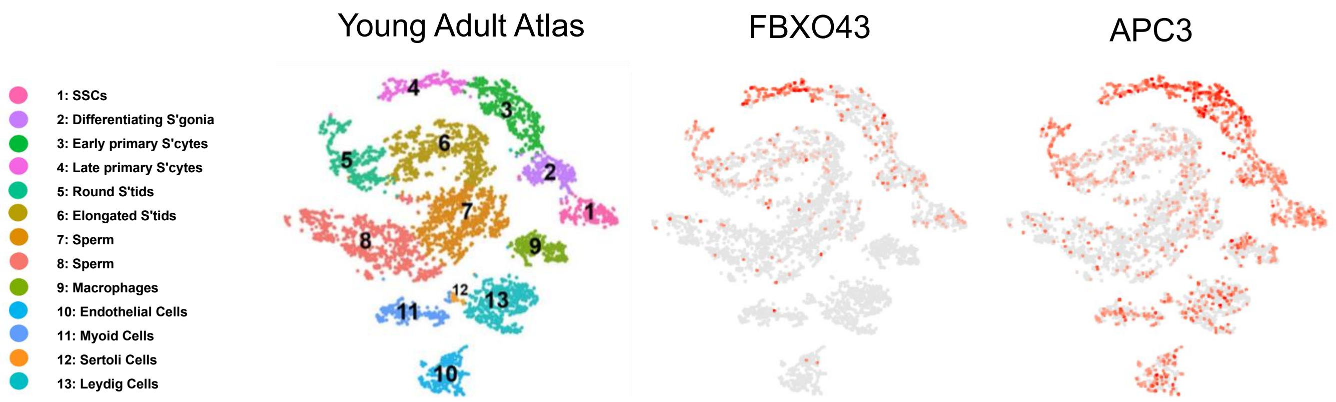

Supplementary Figure 3. Expression profiles of FBXO43 and APC3 across human testicular cell types.

Cell-type-specific expression data were retrieved from the Human Testis Atlas (https://humantestisatlas.shinyapps.io/humantestisatlas1/). FBXO43 is predominantly expressed in germ cells, with prominent enrichment in spermatocytes and round spermatids. APC3 shows a broader distribution, with notable expression also detected in somatic cell types including Sertoli and Leydig cells. Both genes exhibit overlapping enrichment in spermatocytes and round spermatids, supporting their functional relevance in meiotic regulation.
