# Supplementary material for: Mutation Severity of FBXO43 Determines Spermatogenic Outcome and Informs Precision ART Strategies
Source: Hum Mutat. 2026 Aug 12;2026:7905936. doi: 10.1155/humu/7905936 (PMC13469765; doi:10.1155/humu/7905936)
Supplement: Supplementary file 2 [file HUMU-2026-7905936-s001.doc]

Supplementary table. Primers used in this study

| Primer name | Primer sequence (5’-3’) |
| --- | --- |
| FBXO43-WT-F | CCTCTAGCAAGCTCTTCTGTTACTC |
| FBXO43-WT-R | AACACTGCATGGGGGAGTTCT |
| FBXO43-M1-F | AACCTTGCCTAAGGTGCCAGTCCCCTGCTAAGTACCAGC |
| FBXO43-M1-R | GCACCTTAGGCAAGGTTTTAATGCTTCATCAGTAAAAAGTG |
| FBXO43-M2-F | GTGTAGCCAAACAGCCTGTGGTTTTGACTTTTGTGTGTTATG |
| FBXO43-M2-R | AGGCTGTTTGGCTACACAGTCCCCTTTTCTTATATGGCTG |
| FBXO43-M3-F | AAGCAGAAATTAGCGTGAAATTGTTGTTCAAGATAAAAATGCA |
| FBXO43-M3-R | CACGCTAATTTCTGCTTACTTTCCAAACACTGCATAGGCT |
| FBXO43-M4-F | ATCGGCTCAGCTTTAAGATCTGTGCAGGCACAGGCTAGG |
| FBXO43-M4-R | CTTAAAGCTGAGCCGATTTAAAAGCTGGAGCCGAGTGGCA |
| FBXO43-G664D-F | CAGCCTGTGaTTTTGACTTTTGTGTGTTATGTCTGTG |
| FBXO43-G664D-R | GTCAAAAtCACAGGCTGTTCGGCTACACAGTC |

Supplementary table 2. APC/C subunits identified by IP–MS using wild-type FBXO43

| **Gene Symbol (Alias)** | **Protein Name** | **Gene Function** |
| --- | --- | --- |
| ***ANAPC2*** | **APC2** | Forms the catalytic core module of the APC/C together with APC11 as a cullin–RING ligase (CRL). It mediates the interaction with E2 ubiquitin-conjugating enzymes (e.g., UBE2C, UBE2S) and uses its WHB (winged-helix B) domain to engage UBE2C, thereby facilitating ubiquitin transfer. |
| ***ANAPC3 (CDC27)*** | **APC3** | A major tetratricopeptide repeat (TPR) subunit forming the TPR lobe alongside APC6, APC7, and APC8. It interacts with the co-activator and APC10 to assist substrate recruitment. |
| ***ANAPC6 (CDC16)*** | **APC6** | Constitutes the TPR lobe with APC3, APC7, and APC8, forming a structural scaffold essential for APC/C integrity. It participates in co-activator binding and transmits co-activator–dependent signals to the platform domain (APC1–APC4–APC5), inducing conformational remodeling of the complex. |
| ***ANAPC8 (CDC23)*** | **APC8** | Interacts with the co-activator to modulate conformational rearrangements required for substrate recognition. It contributes to assembly of the TPR lobe and provides a structural scaffold for APC/C. |
| ***ANAPC10*** | **APC10** | Cooperates with the co-activator in recognizing D-box (destruction-box) degrons on substrates. It interacts with APC1 and APC3 to maintain APC/C structural stability and promote proper complex assembly. |
| ***ANAPC15*** | **APC15** | Functions as an auxiliary TPR-lobe component. Its interactions with APC8 and APC5 promote TPR-lobe assembly and stabilize the interface between the TPR lobe and the platform domain. |
| ***ANAPC16*** | **APC16** | Regulates the assembly and conformational dynamics of the TPR lobe within the APC/C complex. |
